# Potential for yield and soil fertility improvement with integration of organics in nutrient management for finger millet under rainfed Alfisols of Southern India

**DOI:** 10.3389/fnut.2023.1095449

**Published:** 2023-10-12

**Authors:** Mathyam Prabhakar, Kodigal A. Gopinath, Uppu Sai Sravan, Golla Srasvan Kumar, Merugu Thirupathi, Gutti Samba Siva, Guddad Meghalakshmi, Nakka Ravi Kumar, Vinod Kumar Singh

**Affiliations:** Central Research Institute for Dryland Agriculture, Indian Council of Agricultural Research, Hyderabad, Telangana, India

**Keywords:** finger millet, organic nutrient management, grain yield, nitrogen use efficiency, soil properties

## Abstract

Finger millet (*Eluesine coracana* L.) is gaining importance as a food crop with the increasing emphasis on nutritional aspects and drought resilience. However, the average productivity of the crop has stagnated at around 2,000 kg ha^−1^ in India. Recently released nutrient responsive high yielding varieties are reported to respond better to application of fertilizers/manures. Further, substitution of chemical fertilizers with organic manures to maintain sustainable yields and improve soil health is gaining attention in recent years. Therefore, identifying the appropriate rate and source of nutrition is important to enhance the productivity of finger millet while improving the soil health. A field experiment was conducted during two rainy seasons (July–November, 2018 and 2019) to study the response of finger millet varieties to chemical fertilizers and farmyard manure (FYM) on growth, yields, N use efficiency, N uptake and on soil properties. Two varieties MR-1 and MR-6 were tested with four nutrient management practices *viz.*, unamended control, 100% recommended dose of fertilizers (RDF; 40–20-20 kg NPK ha^−1^), 50% RDF + 50% recommended dose of nitrogen (RDN) as FYM and 100% RDN as FYM. Among the varieties, MR-6 outperformed MR-1 in terms of growth, yield, N use efficiency and N uptake. The yield enhancement was up to 22.6% in MR-6 compared to MR-1 across the nutrient management practices. Substituting FYM completely or half of the fertilizer dose increased the growth and yield of finger millet compared to application of chemical fertilizers alone. Similarly, the average biomass yield, ears m^−2^, grain yield, total N uptake and N use efficiency in response to nutrient management practices followed the order of 100% RDN as FYM > 50% RDF + 50% RDN as FYM > 100% RDF. The soil organic carbon, available N, P, K, and S improved by 25.0, 12.9, 5.7, 6.1, and 22.6%, respectively in the plots under higher rate of FYM application (8 Mg ha^−1^) compared to plots under chemical fertilizers alone. We conclude that substituting chemical fertilizers either completely or by up to 50% with organic manures supplies adequate amounts of nutrients, improves the yield of finger millet, economic returns, and soil properties.

## Introduction

Climate change together with the increasing population is mounting substantial pressure on farming sector to produce more food from less land and depleting natural resources. The productivity of rainfed agriculture constituting about 51% of the cultivated area in India is already constrained by the aberrant monsoon, low and unstable yield, small farm size, degraded soil, and resource poor farmers. Climate change may further severely impact the food production and livelihoods of smallholders in these rainfed areas. One of the possible solutions to counter these tribulations can be identifying and improving native crops that are highly adaptive to local climate, have high nutritive value and can efficiently withstand biotic and/or abiotic stresses (1). Millets can help contribute to some of the biggest global challenges such as nutrition and health needs, climate change mitigation and adaptation, and livelihoods of smallholders particularly in resource-constrained dryland areas. Millets are considered nutritious-cereals due to their high nutritional content, and their potential to address climate change and food security is not entirely realized. The consumption of millets by the people is increasing in recent times due to its nutritional benefits. Moreover, the United Nations General Assembly has declared the year 2023 as the International Year of Millets. Millets are hardy crops, mostly grown under rainfed conditions and perform better even in the low fertile soils (2). These crops require very little water for their cultivation and can be cultivated under rainfed conditions with low rainfall (200–500 mm) (1).

Finger millet (*Eluesine coracana* L.) is a staple food in the arid and semiarid tropics, cultivated in more than 25 countries in Africa and Asia and account for 12% of global millet area (3–5). It is the third most important millet in India next to sorghum and pearl millet. Finger millet is mostly cultivated in arid and semi-arid regions of India and forms an important food particularly to pregnant and lactating mothers, children and diabetic patients due to its better nutritional quality (6). In India, the crop is cultivated in 1.0 million ha with average productivity of 1,747 kg ha^−1^ and the major finger millet growing states are Karnataka, Tamil Nadu, Andhra Pradesh, Odisha, Jharkhand, Maharashtra and Uttarakhand (7, 8). It is a highly productive crop that can thrive under a variety of harsh environmental conditions, and is also organic by default. It can be grown on low fertility soils and is not dependent on the use of chemical fertilizers, hence, is a boon for the vast arid and semi-arid regions (9). The major factors governing the finger millet productivity in the rainfed areas are seasonal rainfall (distribution and amount), soil nutrient status and quantity of fertilizer nutrient applied (10). Indiscriminate use of fertilizers and continuous application of chemical fertilizers has resulted in stagnant yields and declining soil fertility (11). The productivity of finger millet is still low (1,800–2,000 kg ha^−1^) mainly due to major and micronutrient deficiency in the soils and low use of organic manures (12, 13). Hence, balanced nutrient management is key in achieving higher yield and to maintain soil fertility. Further, high cost of chemical fertilizers and their negative impacts on soil health have led to growing interest in the organic nutrient management and conjunctive use of organic and chemical sources of nutrients (6, 14).

The different genotypes of finger millet have genes for early and vigorous growth, large panicle size, increased finger number and branching as well as high-density grains. Some of the genotypes are water-efficient with elevated carbon dioxide fixation rates and minimal leaf area and hence could perform extraordinary well in semi-arid climates. It is also known to be one of the most efficient utilizers of nitrogen (1). Usually, the response of traditional finger millet varieties to fertilizers / nutrient management is less resulting in poor grain yields. The productivity of many short duration varieties (100–110 days) is around 2,500–3,000 kg ha^−1^. However, the two varieties of finger millet (MR-1 and MR-6) which are long duration (120–125 days) but suitable for early rainy (*kharif*) season, have potential to yield 3,500–4,000 kg ha^−1^, and are nutrient responsive under semi-arid tropics. However, studies on integrated nutrient management for finger millet under semi-arid tropics of Southern India are limited. Understanding the response of a particular variety to different nutrient sources will help in determining the nutrient requirement of finger millet varieties. It is important to optimize the nutrient management practice for realizing higher productivity of finger millet under rainfed conditions of semi-arid tropics as the information is scarce. Therefore, the present study was carried out to assess the response of finger millet varieties to different sources of nutrients in terms of crop yield, nitrogen use efficiency and soil properties. Here, the hypothesis we tested was that organic and integrated nutrient management would improve crops yield compared to that of conventional production system due to improvement of soil properties.

## Materials and methods

### Experimental location

**Figure 1:**
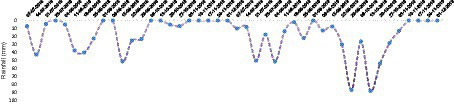
Weekly total rainfall (mm) during the cropping period (July–November) during 2018–2019.

**Figure 2:**
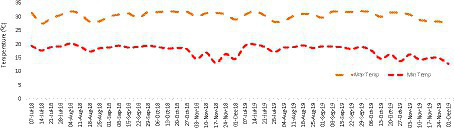
Mean weekly maximum and minimum temperature during the cropping period (July–November) during 2018–2019.

Field studies were carried out for two consecutive rainy seasons, 2018 and 2019 at Gungal Research Farm, ICAR-Central Research Institute for Dryland Agriculture, Telangana, India (17°04′59.94”N longitude, 78°40′01.14″E latitude and at an altitude of 622 above mean sea level) to study the effect of organic manures and chemical fertilizers on finger millet yield and soil properties under rainfed condition. The climate of the region is semi-arid (dry) with demarcated summer (March to May), rainy season (*kharif*) (June to September) and winter (*rabi*) (October to February). The experimental field received 215.9 mm and 517.7 mm rainfall with maximum precipitation in the months of August and October during the crop growth period in 2018 and 2019, respectively. The weakly rainfall and temperature (maximum and minimum) during the crop season (July –November) during the study period (2018 and 2019) are given in Figures 1, 2. The soil of experimental field was sandy clam loam, slightly acidic in reaction (pH 6.2) with electrical conductivity of 0.36 dSm^−1^, low in organic carbon (0.39%), available nitrogen (225.80 kg ha^−1^), medium in available phosphorus (15.60 kg ha^−1^) and available potassium (188.16 kg ha^−1^), sufficient in available sulfur (11.50 kg ha^−1^), iron (5.49 ppm), copper (1.43 ppm) and manganese (16.72 ppm) but deficient in available zinc (0.54 ppm).

### Experimental design and treatment details

The experiment consisted of two factors *viz.*, two varieties (MR-1 and MR-6) and four nutrient management practices *viz.*, unamended control (no fertilizer), 100% recommended NPK (40:20:20 kg ha^−1^), 50% recommended NPK (20:10:10 kg ha^−1^) + farmyard manure (FYM) at 4 Mg ha^−1^, and FYM at 8 Mg ha^−1^. The treatments were combined in 2 × 4 factorial arrangement and the experiment was laid out in a randomized block design with three replications. The experimental field was plowed twice with tractor-drawn cultivator and once with disk harrow, leveled and laid into 5.1 m × 4.2 m plots. MR-1 variety is of long duration (120–125 days to maturity), suitable for early sowing in rainy (*kharif*) season with yield potential of 3,500–4,000 kg ha^−1^. MR-6 variety is also of long duration (120 days to maturity) having tolerance to neck blast and finger blast with yield potential of 3,000–3,500 kg ha^−1^. FYM was sourced from the local farmers near the research farm. Farmers usually prepare FYM by composting crop residues and cattle manure for about four months. On average, FYM had 0.5% N, 0.27% P, 0.4% K, 27.9 ppm Cu, 228.1 ppm Mn, 452 ppm Fe and 143.1 ppm Zn on dry weight basis. FYM was applied on N equivalent basis (dry weight) as per the treatment, i.e., 4 Mg ha^−1^ under 50% FYM and 8 Mg ha^−1^ under 100% FYM treatments. The chemical fertilizers used for the study were urea, diammonium phosphate (DAP) and muriate of potash (MOP). The P and K were applied as basal at the time of sowing. N was applied in two equal splits, half dose at the time of sowing and remaining dose was top dressed at 30 days after sowing (DAS).

### Crop management

Finger millet was sown with the onset of monsoon, i.e., on 19 July 2018 and 06 July 2019. The varieties were sown with a seed rate of 10 kg ha^−1^ and 30 cm row spacing. The chemical fertilizers were broadcasted into the plots as per the treatments and mixed thoroughly with soil before sowing. Well decomposed FYM was applied uniformly in respective plots on dry-weight basis and spread manually three weeks before sowing. Gap filling was done two weeks after sowing to ensure optimum plant population. Weeds were controlled manually after 20 DAS during both rainy seasons. Finger millet was harvested at maturity on 21 November, 2018 and 08 November, 2019 in both seasons. At maturity, the ear heads of finger millet were harvested first and the remaining biomass was harvested manually using a sickle just above the ground (about 5 cm). The ear heads and biomass were sun dried for 2–3 days on a threshing floor and later threshed manually. Grain and stover yields were recorded separately each year after adjusting the seed moisture content to 14%.

### Data collection

Data on growth and yield components of finger millet was collected randomly from tagged/selected plants from an area of 14.04 m^2^ in each plot. At maturity, plant height was measured and above ground biomass was recorded by using destructive sampling. The plants were allowed to shade dry for two days, and later oven dried at 70°C till constant weight was obtained and biomass was recorded. Yield components *viz.*, productive tillers per square meter, number of ears per square meter and fingers per ear head were measured randomly from selected plants (excluding border rows). Ten plants were used for computing fingers per ear head. At maturity, finger millet plants from border rows were discarded to avoid border effect, and ears in each plot were harvested manually, sundried, threshed manually and grain yield was adjusted to 14% moisture. The 1,000 grains from each plot was separated, weighed and expressed as 1,000-grain weight. The remaining stover was harvested plot wise and stover yield was recorded and expressed in kg ha^−1^. The harvest index was calculated as the ratio of grain yield to biological yield and expressed as percentage. The productivity per day was calculated by dividing grain yield with duration of finger millet varieties. The N content in grain and stover were determined by micro Kjeldahl method as described by Jackson (15). The uptake by grain and stover was derived by multiplying respective nutrient contents with their yields.

### Nitrogen use efficiencies

Various parameters of NUE were calculated according to Ju et al. (16)


(1) $$\mathrm{Internal}\;NUE\;\left({\mathrm{IE}}_{N}\right)=\frac{\mathrm{Grain}\;\mathrm{yield}\;\left(\mathrm{kg}\right)}{N\;\mathrm{uptake}\;\left(\mathrm{kg}\right)}$$

(2) $$\begin{array}{c} \mathrm{Apparent}\;\mathrm{recovery}\\ \mathrm{efficiency}\;\left({RE}_{N}\right) \end{array}\;=\frac{\left(\begin{array}{l} N\;\mathrm{uptake}\;\mathrm{in}\;N\;\mathrm{applied}\;\mathrm{plots}\\ -N\;\mathrm{uptake}\;\mathrm{in}\;\mathrm{control}\;\mathrm{plots}\;\left(\mathrm{kg}\right) \end{array}\right)}{N\;\mathrm{rate}\;\left(\mathrm{kg}\right)}\times 100$$

(3) $$\mathrm{Agronomic}\;NUE\;\left({AE}_{N}\right)=\frac{\left(\begin{array}{l} \mathrm{Grain}\;\mathrm{yield}\;\mathrm{in}\;N\;\mathrm{applied}\;\mathrm{plots}\\ -\mathrm{Grain}\;\mathrm{yield}\;\mathrm{in}\;\mathrm{control}\;\mathrm{plots}\;\left(\mathrm{kg}\right) \end{array}\right)}{N\;\mathrm{rate}\;\left(\mathrm{kg}\right)}$$

(4) $$\mathrm{Physiological}\;NUE\;\left({PE}_{N}\right)=\frac{\left(\begin{array}{l} \mathrm{Grain}\;\mathrm{yield}\;\mathrm{in}\;N\;\mathrm{applied}\;\mathrm{plots}\\ -\mathrm{Grain}\;\mathrm{yield}\;\mathrm{in}\;\mathrm{control}\;\mathrm{plots}\;\left(\mathrm{kg}\right) \end{array}\right)}{\left(\begin{array}{l} N\;\mathrm{uptake}\;\mathrm{in}\;N\;\mathrm{applied}\;\mathrm{plots}\\ -N\;\mathrm{uptake}\;\mathrm{in}\;\mathrm{control}\;\mathrm{plots}\;\left(\mathrm{kg}\right) \end{array}\right)}$$

(5) $$\begin{array}{c} \mathrm{Partial}\;\mathrm{factor}\;\mathrm{productivity}\\ \mathrm{of}\;\mathrm{applied}\;\mathrm{nitrogen}\;\left({PFP}_{N}\right) \end{array}\;=\frac{\mathrm{Grain}\;\mathrm{yield}\;\mathrm{in}\;N\;\mathrm{applied}\;\mathrm{plots}\;\left(\mathrm{kg}\right)}{N\;\mathrm{rate}\;\left(\mathrm{kg}\right)}$$

(6) $$\mathrm{Nitrogen}\;\mathrm{harvest}\;\mathrm{index}\;\left({HI}_{N}\right)=\frac{N\;\mathrm{uptake}\;\mathrm{in}\;\mathrm{grains}\;\left(\mathrm{kg}\right)}{N\;\mathrm{uptake}\;\mathrm{in}\;\mathrm{total}\;\mathrm{plant}\;\left(\mathrm{kg}\right)}\times 100$$

### Soil sampling and analysis

In 2018, before the start of the experiment an initial composite soil sample (0–30 cm) was collected and analyzed for different physical and chemical properties. In 2019, after two seasons of experimentation, soil samples from each plot were taken (0–30 cm) for analyzing the chemical properties. Soil organic carbon (SOC) was analyzed using Walkley and Black’s rapid titration method (17), available N by alkaline permanganate method (18), available P by Olsen’s method (19), available potassium by neutral normal ammonium acetate method (15), available S by turbidimetry method (20), micronutrients *viz.*, zinc, iron, copper and manganese by DTPA extraction method (21).

### Economic analysis of crop production

Economic analysis was based on the prevailing cost of input/operations and price of produce. The cost of cultivation involved the expenditure toward land preparation, seed and sowing, manures/mineral fertilizers and their application, harvesting, post-harvest operations, and rental value of land. The farm gate prices of different inputs were considered for economic analysis of crop production. The seed and mineral fertilizer costs were taken from agro-input retailers. Manure can represent a considerable cost to organic producers and can vary widely depending on transport distances and the costs of obtaining the manure (22). However, FYM had no stable market price in our study area and hence it was costed in terms of the labor involved in different activities of composting, loading and transportation within 2 km of the field.

### Data analysis

Statistical methods and tests were performed for different parameters. The crop growth, yield components, yields, nitrogen use efficiencies, soil parameters and economics was tested for the normality and homogeneity of variance using Shapiro Wilk’s test and Bartlett’s test, respectively. The data was normally distributed and homogenous so combined analysis was performed using agricolae package of R software (23). The statistical model used in the analysis included sources of variation due to year, replication, variety, nutrient management, year × variety, year × nutrient management, variety × nutrient management, and year × variety × nutrient management. Tukey’s test was used to compare the treatment means. The *p* < 0.05 was regarded as statistically significant.

## Results

### Growth and yield components

Crop growth and yield components differed significantly between year (except for fingers per head), variety and nutrient management practices (Table 1). Finger millet varieties responded differentially to different rates of chemical fertilizers and organic manures. Variety MR-6 recorded significantly higher plant height, productive tillers m^−2^, above ground biomass, no. of ears m^−2^, fingers ear head^−1^ and 1,000-grain weight than MR-1. Among the nutrient management practices, organic treatment (100% equivalent of N through FYM) enhanced the growth and yield components to a greater extent followed by integrated nutrient management (50% RDF + 50% FYM) and application of chemical fertilizers alone (100% RDF). The biomass and no. of ears m^−2^ increased by 6.0 and 8.3%, respectively with application of 100% FYM compared to 100% RDF. Fingers per ear head were higher by 13.0–20.7% with application of organic and chemical fertilizers compared to control.

**Table 1:** Growth and yield attributes of finger millet as influenced by varieties and nutrient management (pooled across two rainy seasons, 2018 and 2019).

Treatment	Plant height (cm)	Productive tillers m^−2^	Above ground biomass (g plant^−1^)	No. of ears m^−2^	Fingers ear head^−1^
*Variety (V)*					
V_1_: MR-1	104.9^b^	92^b^	25.1^b^	258^b^	5.5^b^
V_2_: MR-6	112.8^a^	100^a^	28.2^a^	272^a^	6.4^a^
*Nutrient management (NM)*					
F_1_: Unamended control	103.9^b^	88^b^	19.9^c^	227^c^	5.3^b^
F_2_: 100% RDF	109.4^a^	97^a^	28.1^b^	266^b^	6.1^a^
F_3_: 50% RDF + 50% FYM	110.3^a^	98^a^	28.9^ab^	277^ab^	6.0^a^
F_4_: 100% FYM	111.8^a^	100^a^	29.9^a^	290^a^	6.4^a^

Source	Pr > F and significance				
Y	0.0197^*^	<0.0001^**^	0.0272^*^	0.0230^*^	0.1181^ns^
V	<0.0001^**^	<0.0001^**^	<0.0001^**^	0.0354^*^	<0.0001^**^
NM	0.0009^**^	<0.0001^**^	<0.0001^**^	<0.0001^**^	0.0008^**^
Y × V	0.8238^ns^	0.1514^ns^	0.7523^ns^	0.7243^ns^	0.4953^ns^
Y × NM	0.8602^ns^	0.8816^ns^	0.6447^ns^	0.9506^ns^	0.8992^ns^
V × NM	0.6765^ns^	0.0255^*^	0.0210^*^	0.7494^ns^	0.2857^ns^
Y × V × NM	0.9823^ns^	0.9334^ns^	0.5449^ns^	0.8570^ns^	0.3335^ns^

Recommended dose of fertilizer (RDF): 40:20:20 kg ha^−1^; Mean values followed by different letters within each column denote significance at *p* < 0.05, Tukey’s test. ^*^ and ^**^ indicate the significance levels at *p* < 0.05 and 0.01 respectively, ^ns^ non-significant at *p* > 0.05.

The interaction between variety and nutrient management showed significant difference for productive tillers m^−2^, above ground biomass and 1,000-grain weight (Table 1). The tillers, biomass and 1,000-grain weight of varieties MR-6 and MR-1 was similar at 100% FYM and 50% RDF + 50% FYM. The variety MR-6 produced better yield components than MR-1 at the same nutrient management practice (Table 2). The maximum tillers and crop biomass were obtained with 100% RDF for MR-6 whereas 1,000-grain weight was maximum with 100% FYM for MR-6.

**Table 2:** Interaction effect of varieties and nutrient management on growth, yield attributes and yield of finger millet.

Treatment		Productive tillers m^−2^	Biomass (g plant^−1^)	1,000-grain weight (g)	Grain yield (kg ha^−1^)	Biological yield (kg ha^−1^)	Productivity per day (kg ha^−1^)
MR-1	Unamended control	81^c^	18.8^d^	1.77^c^	1444^c^	6194^d^	11.6^e^
	100% RDF	91^b^	25.2^c^	1.82^ab^	2393^d^	8300^c^	19.1^d^
	50% RDF + 50% FYM	96^ab^	27.5^bc^	1.81^bc^	2780^c^	9082^bc^	22.2^bc^
	100% FYM	98^ab^	28.9^ab^	1.82^ab^	2937^bc^	9534^ab^	23.5^bc^
MR-6	Unamended control	95^ab^	20.9^d^	1.81^bc^	1754^c^	6908^d^	14.0^e^
	100% RDF	103^a^	30.9^a^	1.82^ab^	3384^a^	10217^a^	27.1^a^
	50% RDF + 50% FYM	101^a^	30.2^ab^	1.86^a^	3267^ab^	9971^ab^	26.1^ab^
	100% FYM	102^a^	30.8^a^	1.87^a^	3316^a^	10172^a^	26.5^a^

Recommended dose of fertilizer (RDF): 40:20:20 kg ha^−1^; Mean values followed by different letters within each column denote significance at *p* < 0.05.

### Crop yield

Finger millet yields (grain, stover and biological) varied significantly with both varieties and nutrient management (Table 3). However, significant differences with year was noted for grain and biological yields only. Among varieties, MR-6 produced 22.6, 8.5 and 12.6% higher grain, straw and biological yields compared to MR-1. The grain, stover and biological yields of finger millet with application of two rates of FYM was better than that of chemical fertilizers alone. Highest rate of FYM application (8 Mg ha^−1^) consistently resulted in better yields, followed by combined application of chemical fertilizer and FYM over chemical fertilizers alone and unamended control. Application of 100% FYM produced 95.6, 35.8 and 50.4% more grain, straw and biological yields, respectively over unamended control. Organic nutrient management (100% FYM) produced 8.2 and 6.4% more grain and biological yields, respectively over application of chemical fertilizers alone (100% RDF).

**Table 3:** Thousand-grain weight, yields, harvest index, and productivity per day of finger millet as influenced by varieties and nutrient management (pooled across two rainy seasons, 2018 and 2019).

Treatment	1,000-grain weight (g)	Grain yield (kg ha^−1^)	Stover yield (kg ha^−1^)	Biological yield (kg ha^−1^)	Harvest index (%)	Productivity per day (kg ha^−1^)
*Variety (V)*						
V_1_: MR-1	1.81^b^	2389^b^	5889^b^	8278^b^	28.4^b^	19.1^b^
V_2_: MR-6	1.84^a^	2930^a^	6387^a^	9317^a^	31.0^a^	23.4^a^
*Nutrient management (NM)*						
F_1_: Unamended control	1.79^b^	1599^c^	4953^b^	6551^c^	24.4^b^	12.8^c^
F_2_: 100% RDF	1.82^a^	2889^b^	6370^a^	9259^b^	31.0^a^	23.1^b^
F_3_: 50% RDF + 50% FYM	1.83^a^	3023^ab^	6503^a^	9526^ab^	31.7^a^	24.2^ab^
F_4_: 100% FYM	1.84^a^	3127^a^	6727^a^	9853^a^	31.8^a^	25.0^a^

Source	Pr > F and significance					
Y	<0.0001^**^	<0.0139^*^	0.1589^ns^	0.0272^*^	0.4741^ns^	0.0022^**^
V	<0.0001^**^	<0.0001^**^	0.0015^**^	<0.0001^**^	0.0009^**^	<0.0001^**^
NM	<0.0001^**^	<0.0001^**^	<0.0001^**^	<0.0001^**^	<0.0001^**^	<0.0001^**^
Y × V	0.1618^ns^	0.8821^ns^	0.6895^ns^	0.7523^ns^	0.7291^ns^	0.8234^ns^
Y × NM	0.6433^ns^	0.7596^ns^	0.8055^ns^	0.6447^ns^	0.9860^ns^	0.6923^ns^
V × NM	0.0322^*^	0.0009^**^	0.3751^ns^	0.0210^*^	0.6279^ns^	0.0009^**^
Y × V × NM	0.6557^ns^	0.6702^ns^	0.5422^ns^	0.5449^ns^	0.6349^ns^	0.6597^ns^

Recommended dose of fertilizer (RDF): 40:20:20 kg ha^−1^; Mean values followed by different letters within each column denote significance at *p* < 0.05, Tukey’s test. ^*^ and ^**^ indicate the significance levels at *p* < 0.05 and 0.01 respectively, ^ns^ non-significant at *p* > 0.05.

The interaction effect of varieties and nutrient management practices showed significant differences for grain and biological yield. Varieties showed differential response with nutrient management practices, MR-6 performed better with chemical fertilizers while MR-1 performed better under organic nutrient management (Table 2). Yield obtained under MR-6 with 100% RDF (3,384 kg ha^−1^) was slightly better than that under 100% FYM (3,316 kg ha^−1^). Application of 100% RDF to MR-6 resulted in 41.4% more yield compared to MR-1 at same dose. The response of biological yield to different treatments was similar as that of grain yield. Overall, MR-1 registered lower grain and biological yields compared to MR-6 at all the nutrient management practices.

### Harvest index and productivity per day

Harvest index varied with varieties and nutrient management practices, whereas productivity per day was affected due to year, varieties, nutrient management and interaction of varieties and nutrient management practices (Table 3). Performance of variety MR-6 was better than MR-1 for both harvest index and productivity per day. Different nutrient management treatments had similar but significantly higher harvest index compared to unamended control. Our study revealed that application of 100% FYM produced 1.9 kg ha^−1^ more productivity per day than 100% RDF across both varieties. Interaction effect showed that productivity per day of MR-1 ranged from 19.1–23.5 kg ha^−1^ whereas that of MR-6 ranged from 26.1–27.1 kg ha^−1^ under different nutrient management treatments (Table 2). This clearly shows that MR-6 was better compared to MR-1.

### Nitrogen use efficiencies

**Figure 3:**
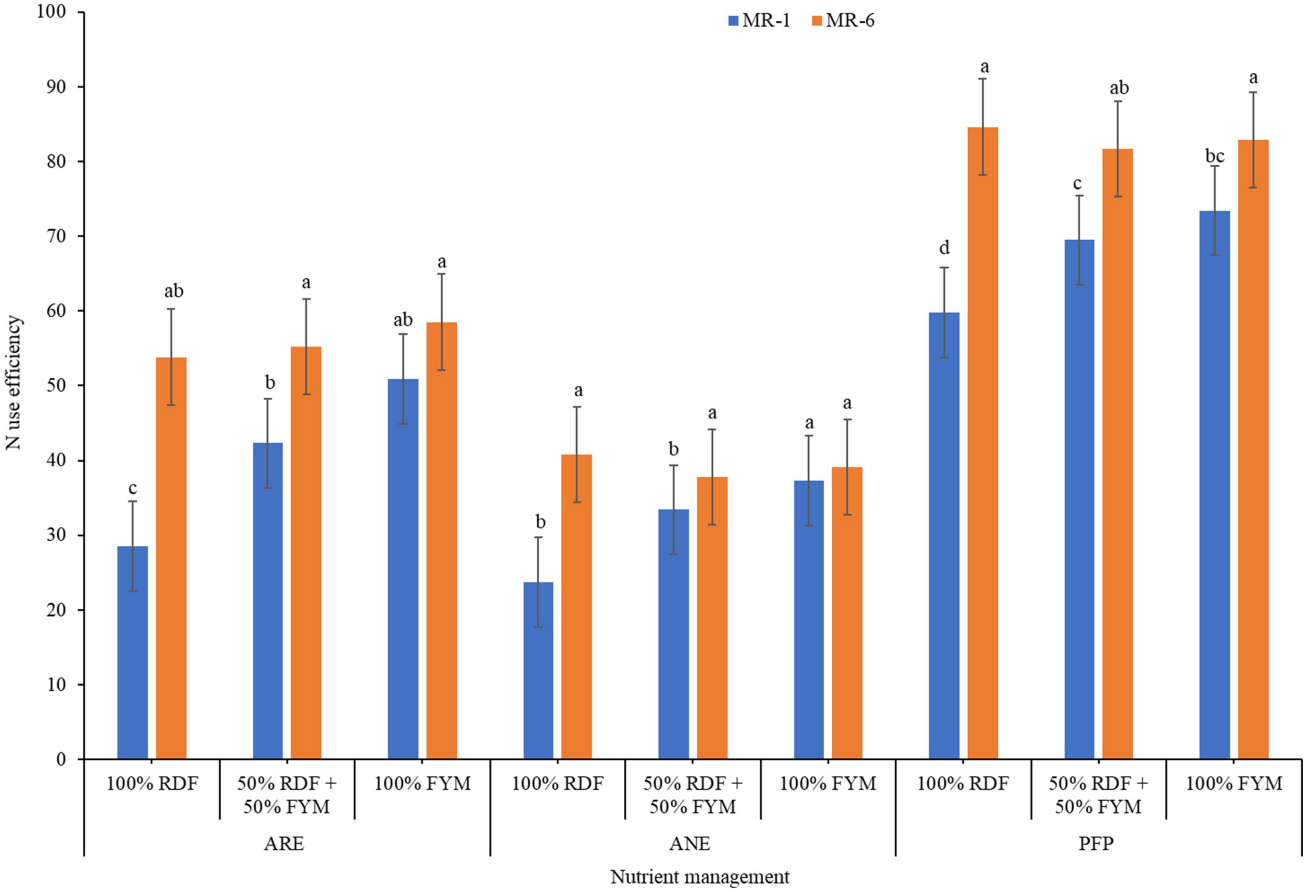
Interaction effect of varieties and nutrient management on apparent recovery efficiency (AR_E_), agronomic N use efficiency (AN_E_), and partial factor productivity (PFP) of finger millet.

Internal N use efficiency (N utilization efficiency) did not alter either with varieties or nutrient management practices (Table 4). Apparent recovery efficiency (N uptake efficiency) and agronomic N use efficiency (N use efficiency) varied significantly with varieties, nutrient management practices and their interaction (Table 4). Among varieties, MR-6 was better with 15.2 and 7.7% higher apparent recovery efficiency and agronomic N use efficiency, respectively compared to MR-1. Plots receiving 100% FYM recorded 13.6 and 5.9% higher apparent recovery efficiency and agronomic N use efficiency, respectively over 100% RDF. The N uptake efficiency of both varieties was improved with 100% FYM, whereas N use efficiency response varied for varieties (Figure 3). N use efficiency of MR-1 was maximum (37.3 kg kg^−1^) with 100% FYM, whereas for MR-6 it was maximum with 100% RDF (40.8 kg kg^−1^). N uptake efficiency of MR-6 ranged from 53.7–58.5% while it was 28.5–50.9% for MR-1 with different nutrient management practices. N use efficiency ranged from 37.8–40.8% in MR-6 and 23.7–37.3% in MR-1 under different nutrient management practices (Figure 3). Physiological N use efficiency significantly varied with varieties only, and maximum efficiency with MR-1. Partial factor productivity showed significant differences with varieties, nutrient management practices and their interaction (Table 4). The response of partial factor productivity (PFP) was similar to that of N use efficiency. Variety MR-6 under 100% RDF had higher PFP (84.6 kg kg^−1^), whereas MR-1 under 100% FYM enhanced PFP (73.4 kg kg^−1^). N harvest index (NHI) differed with nutrient management practices only, maximum improvement was noticed under 50% RDF + 50% FYM (7.7%) compared to unamended control.

**Table 4:** Nitrogen use efficiencies of finger millet as influenced by varieties and nutrient management (pooled across two rainy seasons, 2018 and 2019).

Treatment	Internal N use efficiency (kg kg^−1^) IE_N_	Apparent recovery efficiency (%) AR_E_	Agronomic N use efficiency (kg kg^−1^) AN_E_	Physiological N use efficiency (kg kg^−1^) PN_E_	Partial factor productivity (kg kg^−1^) PFP	N harvest index (%) NHI
*Variety (V)*						
V_1_: MR-1	68.8^a^	40.6^b^	31.5^b^	58.1^a^	50.7^b^	61.0^a^
V_2_: MR-6	69.7^a^	55.8^a^	39.2^a^	53.0^b^	62.3^a^	62.8^a^
*Nutrient management (NM)*						
F_1_: Unamended control	67.0a	–	–	–	–	56.4^b^
F_2_: 100% RDF	71.6^a^	41.1^b^	32.3^b^	78.7^a^	72.2^b^	63.9^a^
F_3_: 50% RDF + 50% FYM	69.9^a^	48.8^a^	35.6^ab^	74.3^a^	75.6^ab^	64.1^a^
F_4_: 100% FYM	68.6^a^	54.7^a^	38.2^a^	70.5^a^	78.2^a^	63.3^a^

Source	Pr > F and significance					
Y	0.229^ns^	0.0612^ns^	0.2549^ns^	0.7991^ns^	0.0215^*^	0.9206^ns^
V	0.517^ns^	<0.0001^**^	0.0004^**^	0.0292^*^	<0.0001^**^	0.0838^ns^
NM	0.131^ns^	<0.0001^**^	0.0481^*^	0.1416^ns^	0.0347^*^	<0.0001^**^
Y × V	0.715^ns^	0.9344^ns^	0.2753^ns^	0.2537^ns^	0.8643^ns^	0.5170^ns^
Y × NM	0.969^ns^	0.3312^ns^	0.7371^ns^	0.8429^ns^	0.7096^ns^	0.9824^ns^
V × NM	0.353^ns^	0.0134^*^	0.0061^**^	0.8313^ns^	0.0037^**^	0.7291^ns^
Y × V × NM	0.771^ns^	0.7113^ns^	0.6229^ns^	0.8845^ns^	0.5876^ns^	0.5417^ns^

Recommended dose of fertilizer (RDF): 40:20:20 kg ha^−1^; Mean values followed by different letters within each column denote significance at *p* < 0.05, Tukey’s test. ^*^ and ^**^ indicate the significance levels at *p* < 0.05 and 0.01 respectively, ^ns^ non-significant at *p* > 0.05.

### Nitrogen content and uptake

Except for grain N content, rest of the parameters *viz.*, stover N content, N uptake by grain, stover and total plant uptake showed significant variations with varieties and nutrient management practices (Table 5). Varietal difference for grain N content was not observed but stover N content was significantly higher in MR-6 than in MR-1. Nutrient management practices had significant differences for both grain and stover N content. Growing finger millet with complete organics (100% FYM) improved N content in grain and stover by 3.4 and 8.7%, respectively compared to 100% RDF.

**Table 5:** Nitrogen content and uptake of finger millet as influenced by varieties and nutrient management (pooled across two rainy seasons, 2018 and 2019).

Treatment	Grain N content (%)	Stover N content (%)	Grain N uptake (kg ha^−1^)	Stover N uptake (kg ha^−1^)	Total N uptake (kg ha^−1^)
*Variety (V)*					
V_1_: MR-1	0.89^a^	0.22^b^	21.3^b^	13.2^b^	34.5^b^
V_2_: MR-6	0.90^a^	0.24^a^	26.7^a^	15.4^a^	42.0^a^
*Nutrient management (NM)*					
F_1_: Unamended control	0.84^b^	0.21^c^	13.4^c^	10.4^c^	23.8^c^
F_2_: 100% RDF	0.89^a^	0.23^bc^	25.8^b^	14.4^b^	40.3^b^
F_3_: 50% RDF + 50% FYM	0.91^a^	0.24^ab^	27.8^a^	15.5^ab^	43.3^a^
F_4_: 100% FYM	0.92^a^	0.25^a^	28.9^a^	16.8^a^	45.7^a^

Source	Pr > F and significance				
Y	0.108^ns^	0.0475^*^	0.0007^**^	0.0086^**^	<0.0001^**^
V	0.190^ns^	0.0052^**^	<0.0001^**^	<0.0001^**^	<0.0001^**^
NM	<0.0001^**^	<0.0001^**^	<0.0001^**^	<0.0001^**^	<0.0001^**^
Y × V	0.696^ns^	0.7521^ns^	0.6556^ns^	0.6736^ns^	0.944^ns^
Y × NM	0.747^ns^	0.9723^ns^	0.3259^ns^	0.6566^ns^	0.180^ns^
V × NM	0.425^ns^	0.0656^ns^	0.0005^**^	0.0310^*^	<0.0001^**^
Y × V × NM	0.980^ns^	0.8576^ns^	0.6906^ns^	0.5141^ns^	0.779^ns^

Recommended dose of fertilizer (RDF): 40:20:20 kg ha^−1^; Mean values followed by different letters within each column denote significance at p < 0.05, Tukey’s test. ^*^ and ^**^ indicate the significance levels at *p* < 0.05 and 0.01 respectively, ^ns^ non-significant at *p* > 0.05.

**Figure 4:**
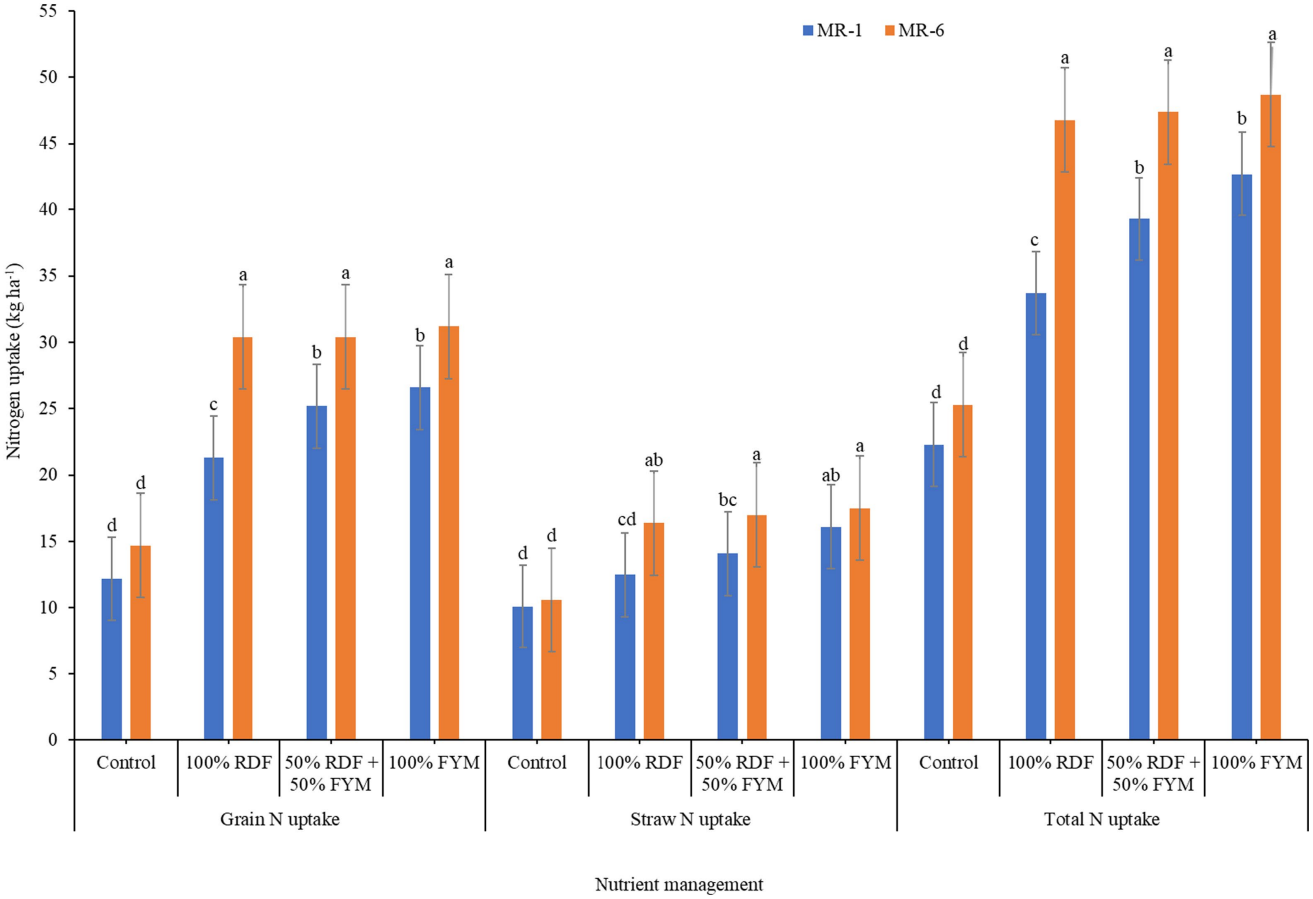
Interaction effect of varieties and nutrient management on grain, stover and total nitrogen uptake of finger millet.

Consequently, plant N uptake variations were also noticed with nutrient management practices for both varieties (Figure 4). Variety MR-6 responded better to different organics and chemical fertilizers with higher nutrient uptake compared to MR-1. Uptake of N by grain and stover of MR-6 variety was enhanced by 25.4 and 16.7%, respectively compared to MR-1. Obviously, total plant N uptake was also greater with MR-6 than MR-1. N uptake by different plant portions (grain and stover) was improved to the maximum extent with application of 8 Mg ha^−1^ FYM followed by combined application of organics and chemical fertilizers (4 Mg ha^−1^ FYM + 50% RDF). Plots receiving FYM either completely or partially resulted in improved total N uptake (7.4–13.4%) compared to application of chemical fertilizers alone.

### Soil properties

In this two-year study under rainfed conditions, both varieties and nutrient management practices had significant impact on soil properties (Table 6). Finger millet varieties had significant effects on available N, P, K, S, and Zn whereas no significant difference was noted for SOC and DTPA extractable Fe, Cu, and Mn. Among the two varieties, MR-1 improved the soil properties better compared to MR-6. Plots under FYM (8 Mg ha^−1^) improved the soil properties to a great extent followed by FYM at 4 Mg ha^−1^. SOC, available N, P, K, and S improved in the range of 2.6–15.4%, 6.0–13.3%, 6.9–9.6%, 5.8–9.8%, and 25.9–47.7%, respectively compared to the initial status with application of different rates of FYM. Higher application rate of FYM (8 Mg ha^−1^) enhanced micronutrients availability at the end of 2-years by 61.1, 11.4, 10.5, and 19.1% for Zn, Fe, Cu, and Mn, respectively over their initial status.

**Table 6:** Soil properties as influenced by varieties and nutrient management after two years.

Treatment	SOC (%)	Available N (kg ha^−1^)	Available P_2_O_5_ (kg ha^−1^)	Available K_2_O (kg ha^−1^)	Available S (ppm)	DTPA extractable Zn (ppm)	DTPA extractable Fe (ppm)	DTPA extractable Cu (ppm)	DTPA extractable Mn (ppm)
*Variety (V)*									
V_1_: MR-1	0.39^a^	241.47^a^	16.55^a^	201.80^a^	15.70^a^	0.71^a^	5.67^a^	1.47^a^	17.45^a^
V_2_: MR-6	0.38^a^	226.31^b^	16.24^b^	189.22^b^	12.47^b^	0.61^b^	5.45^a^	1.44^a^	17.00^a^
*Nutrient management (NM)*									
F_1_: Unamended control	0.33^c^	215.34^c^	15.64^d^	181.79^b^	11.00^b^	0.53^c^	4.95^b^	1.35^a^	13.84^c^
F_2_: 100% RDF	0.36^bc^	225.79^bc^	16.17^c^	194.66^ab^	13.86^ab^	0.56^c^	5.17^ab^	1.42 ^a^	16.32^b^
F_3_: 50% RDF + 50% FYM	0.40^ab^	239.38^ab^	16.67^b^	199.09^a^	14.48^ab^	0.67^b^	6.01^a^	1.49^a^	18.84^a^
F_4_: 100% FYM	0.45^a^	255.06^a^	17.10^a^	206.51^a^	16.99^a^	0.87^a^	6.12^a^	1.58^a^	19.92^a^

Source	Pr > F and significance								
V	0.733^ns^	0.034^*^	0.002^**^	0.029^**^	0.024^**^	0.003^**^	0.487^ns^	0.581^ns^	0.400^ns^
NM	0.019^*^	0.004^**^	<0.001^**^	0.029^**^	0.037^**^	<0.001^**^	0.042^*^	0.050^ns^	<0.001^**^
V × NM	0.982^ns^	0.321^ns^	0.757^ns^	0.266^ns^	0.654^ns^	0.054^ns^	0.993^ns^	0.502^ns^	0.952^ns^
Initial status	0.39	225.80	15.60	188.16	11.50	0.54	5.49	1.43	16.72

Recommended dose of fertilizer (RDF): 40:20:20 kg ha^−1^; Mean values followed by different letters within each column denote significance at *p* < 0.05, Tukey’s test. ^*^ and ^**^ indicate the significance levels at *p* < 0.05 and 0.01 respectively, ^ns^ non-significant at *p* > 0.05.

### Economics of finger millet cultivation

Among the varieties, MR-6 gave higher net returns (Rs. 62,797 ha^−1^) and benefit cost ratio (1.94) than that of MR-1. In general, the cost of finger millet cultivation was higher for the treatments involving FYM. Application of 100% equivalent N through FYM had highest cost of cultivation (Rs. 39,033 ha^−1^) and gross returns (Rs. 101,410 ha^−1^) than other treatments. However, application of chemical fertilizers alone (100% RDF) gave higher net returns (Rs. 64,739 ha^−1^) and benefit cost ratio (2.23) than other treatments (Table 7). The interaction effect of varieties and nutrient management practices showed significant differences for economics of finger millet cultivation. MR-1 variety with application of 100% equivalent N through FYM gave higher net returns (Rs. 56,572 ha^−1^) than other treatments (Table 8). However, in MR-6, application of chemical fertilizers alone (100% RDF) gave higher net returns (Rs. 80,237 ha^−1^) and benefit cost ratio (2.77) than other treatments.

**Table 7:** Economics of finger millet as influenced by varieties and nutrient management (pooled across two rainy seasons, 2018 and 2019).

Treatment	Cost of cultivation (Rs ha^−1^)	Gross returns (Rs ha^−1^)	Net returns (Rs. ha^−1^)	Benefit cost ratio
*Variety (V)*				
V_1_: MR-1	32,284	78196^b^	45912^b^	1.40^b^
V_2_: MR-6	32,284	95081^a^	62797^a^	1.94^a^
*Nutrient management (NM)*				
F_1_: Unamended control	27,033	53344^c^	26311^b^	0.97^d^
F_2_: 100% RDF	29,035	93774^bc^	64739^a^	2.23^a^
F_3_: 50% RDF + 50% FYM	34,034	98025^ab^	63992^a^	1.88^b^
F_4_: 100% FYM	39,033	101410^a^	62377^a^	1.59^c^

Source	Pr > F and significance			
Y	–	0.0023^**^	0.0085^**^	0.3950^ns^
V	–	<0.0001^**^	<0.0001^**^	<0.0001^**^
NM	–	<0.0001^**^	<0.0001^**^	<0.0001^**^
Y × V	–	0.6137^ns^	0.6137^ns^	0.9133^ns^
Y × NM	–	0.3304^ns^	0.3304^ns^	0.6133^ns^
V × NM	–	0.0007^**^	0.0007^**^	0.0002^**^
Y × V × NM	–	0.6035^ns^	0.6035^ns^	0.7805^ns^

Recommended dose of fertilizer (RDF): 40:20:20 kg ha^−1^; Mean values followed by different letters within each column denote significance at *p* < 0.05, Tukey’s test. ^*^ and ^**^ indicate the significance levels at *p* < 0.05 and 0.01 respectively, ^ns^ non-significant at *p* > 0.05; One US $ = Rs 80.9.

**Table 8:** Interaction effect of varieties and nutrient management on economics of finger millet.

Treatment		Gross returns (Rs ha^−1^)	Net returns (Rs ha^−1^)	Benefit cost ratio
MR-1	Unamended control	48417^bC^	21383^bC^	0.79^bC^
	100% RDF	78276^bB^	49241^bB^	1.70^bA^
	50% RDF + 50% FYM	90487^bA^	56453^bA^	1.66^bAB^
	100% FYM	95605^bA^	56572^bA^	1.45^bB^
MR-6	Unamended control	58271^aB^	31238^aC^	1.16^aD^
	100% RDF	109272^aA^	80237^aA^	2.77^aA^
	50% RDF + 50% FYM	105564^aA^	71530^aB^	2.10^aB^
	100% FYM	107216^aA^	68183^aB^	1.75^aC^

Recommended dose of fertilizer (RDF): 40:20:20 kg ha^−1^; Mean value followed by different lower-case letters within each column denote significance at *p* < 0.05, Tukey’s test of varieties at nutrient management and upper-case letters within each column denote significance at *p* < 0.05, Tukey’s test of nutrient management at varieties; One US $ = Rs 80.9.

## Discussion

Although finger millet is reported to be less responsive to higher doses of nutrients, recently developed varieties are both nutrient responsive and high yielding than traditional varieties. Finger millet varieties (MR-6 and MR-1) responded differentially to different rates of chemical fertilizers and organic manures. In general, variety MR-6 registered belter growth and yield attributes than that of MR-1. Differential response of varieties is obvious as they differ genetically and also respond differently to the fertilizers/manures applied (14). The differences in plant height of finger millet due to varieties was also reported by Chohan et al. (24). These improved varieties of finger millet responded well to the applied fertilizers/manures and resulted in better yields. Similarly, earlier studies also reported differential response in terms of growth and yield attributes of finger millet varieties to different fertilizer doses (25, 26). It is evident that the yield differences between varieties was mainly due to their genetic potential and also due to higher tillers, ears m^−2^ and fingers ear head^−1^. Similar varietal differences in grain yield were also reported by Triveni et al. (27).

Balanced application of nutrients is a pre-requisite to exploit the full genetic potential of any crop. Among the nutrient management practices, organic treatment (100% equivalent of N through FYM) enhanced the growth and yield components to the maximum extent followed by integrated nutrient management (50% RDF + 50% FYM) and application of chemical fertilizers alone (100% RDF). Organic manures supply macro-nutrients as well as micronutrients, and release them slowly throughout the crop growth period, help in acceleration of various metabolic processes leading to better crop performance (28). The biomass and no. of ears m^−2^ increased by 6.0 and 8.3%, respectively with application of 100% FYM compared to 100% RDF. Sunitha et al. (29) and Maitra et al. (30) also reported similar finger millet tiller number with chemical fertilizer and integrated nutrient management. Earlier findings also suggested that FYM application at 7.5–10.0 Mg ha^−1^ resulted in better growth and development including root growth (31). Fingers per ear head were higher with application of organic and chemical fertilizers compared to control. With the fertilizer and manure application, tillering was improved that might have resulted in greater interception of photosynthetically active radiation and enhanced crop photosynthesis. Such instances of improvement of yield attributes with application of organics and chemical fertilizers was also observed by other researchers (6, 32, 33).

The grain, stover and biological yields of finger millet were better with application of FYM than that of chemical fertilizers alone. Highest rate of FYM application (8 Mg ha^−1^) consistently resulted in better yields, followed by combined application of chemical fertilizer and FYM over chemical fertilizers alone and unamended control. The improved yields were mainly due to enhanced nutrient supply from FYM. FYM application supplied the required amounts of macro- and micronutrients needed for finger millet and better retention of soil moisture during the entire crop growth period. These results were in corroboration with Prashanth et al. (34). Supplementation of nutrients through organic manures enhanced nutrient availability throughout the crop season that resulted in better growth and development of crop and increased the crop productivity (35). The increased uptake of nutrients helps in accumulation and translocation of these nutrients to developing ear heads, making better filling of ear heads and grain weight leading to higher yields (36). Further, application of higher rate of organic manures reduces soil water evaporation, improves soil moisture holding capacity and supply of macro- and micronutrients (37). Similarly, balanced supply of nutrients improved growth, tillering and better leaf area resulting in higher stover yields (38). Complete supplementation of nutrients through organics (100% FYM) proved better than other treatments in both seasons. Similar response of finger millet to different organics and chemical fertilizers were also reported by Prashanth et al. (34).

Performance of variety MR-6 was better than MR-1 for both harvest index and productivity per day. Varietal differences for harvest index was also reported by Wafula et al. (39). Different nutrient management treatments had similar but significantly higher harvest index compared to unamended control. Differences in harvest index of finger millet to varied supply of organic and chemical fertilizers was reported by Chowdary and Patra (40). Mineral nutrition has a major role in influencing the harvest index (41). Our study revealed that application of 100% FYM resulted in 1.9 kg ha^−1^ more productivity per day than 100% RDF across both varieties. Better yields and shorter crop duration enhanced the productivity per day. Similar observations were also reported by Triveni et al. (27).

Plants either try to increase the amount of N they acquire from soil or use the N efficiently which they have taken up (42). It is beneficial if the crop has both or either of the traits under limited nitrogen conditions. Differential response of various nitrogen use efficiencies in finger millet for both varieties and nitrogen rates were also reported by Gupta et al. (43). Our results showed that MR-6 variety grown with 100% FYM produced greater yields and uptake, thus exhibiting higher N uptake efficiency and N use efficiency, as N uptake is most important factor governing yield (44). Thus, MR-6 would be better variety compared to MR-1 without sacrificing grain yield potential.

Varietal difference for grain N content was not observed but stover N content was significantly higher in MR-6 than in MR-1. The differential N content between varieties might be due to their genetic character and environmental conditions (45). Similar findings of differential response of varieties in N content was also reported by Wafula et al. (39). Growing finger millet with complete organics (100% FYM) improved N content in grain and stover by 3.4 and 8.7%, respectively compared to 100% RDF. The improved N contents may be due to enhanced supply of nutrients to crop that might have resulted in better absorption of N from soil. The synergistic effect of chemical fertilizers and organics might have supplied nutrients at faster pace and continuous release of N from mineralization of organic sources. Improved N content of finger millet with organics and chemical fertilizers was also reported by Jagathjothi et al. (46).

Differential uptake pattern with varieties could be due to the variations in aboveground biomass and their respective N contents. Wafula et al. (39) also found differences in varietal N accumulations. Plots receiving FYM either completely or partially resulted in improved total N uptake (7.4–13.4%) compared to application of chemical fertilizers alone. This positive effect of FYM might be due to solubilization of native nutrients during the decomposition of organic manures, their mobilization and accumulation in different plant parts (47). Lower N accumulation by finger millet in unamended control could be due to lower biomass and lesser available nutrients in soil (34).

Both varieties and nutrient management had significant impact on soil properties (Table 6). Among the two varieties, MR-1 improved the soil properties marginally better compared to MR-6. Among nutrient management treatments, plots under higher dose of FYM (8 Mg ha^−1^) improved the soil properties to a great extent followed by FYM at 4 Mg ha^−1^. Improvement in organic C with application of FYM could be attributed to higher rate of C addition to soil, better plant growth and activity of microorganisms. Similar results of soil health improvement with long-term use of manure and fertilizer were also reported under semi-arid tropics by Vineela et al. (48) and Srinivasarao et al. (49, 50). Further, improvement in available N might be due to increased soil organic C and soil microbial biomass C, better biological activities and release of N through decomposition of organic manures (51, 52). The improvement of available P is possibly due to mineralization of organic P and solubilization of soil P by release of organic acids produced during decomposition of organic matter from FYM. Such improvement in available P with application of FYM was also reported by Roy et al. (53). FYM contains high amounts of K and its application also aids in minimizing the leaching loss of K, and enhancing the solubility of K compounds during the decomposition process. This increase in K availability with FYM application may be attributed to decomposition of primary minerals by carbonic acid and release of nutrients. Buildup of K with application of FYM was also reported by Jaskulska et al. (54). Macronutrients (NPK) availability in soil was increased with application of FYM (55). The improvement of micronutrients availability in soil with application of FYM alone and in combination with chemical fertilizers could be due to build-up of organic matter and these elements form stable complexes with organic ligands resulting in less susceptibility to adsorption and fixation in the soil (56). Similarly, improvement in DTPA extractable Zn, Mn, Fe, and Cu availability in soil with application of FYM was also observed by Chaudhary and Narwal (57), Antil and Singh (58). Similar positive effects on soil chemical properties with application of FYM alone and in combination with chemical fertilizers were reported by Satish et al. (59).

Net returns and benefit cost ratio differed among different treatments. Manure can represent a substantial cost to organic producers and can vary widely depending on transport distances and the costs of obtaining the manure (22). We also observed significantly higher production costs for treatments involving FYM. As a result, the net returns and benefit cost ratio were lower for the treatments involving FYM.

## Conclusion

Improved understanding of response of finger millet varieties to nutrient management is important to develop suitable nutrient management recommendations for rainfed areas of Southern India. Application of organic manures such as FYM enhanced the finger millet growth, and grain yield compared to application of chemical fertilizers alone and unamended control. Finger millet variety MR-6 produced 22.6% higher grain yield compared to MR-1 across the nutrient management practices. Further, varieties showed differential response to nutrient management practices, MR-6 performed better with chemical fertilizers while MR-1 performed better under organic nutrient management. Among nutrient management treatments, organic nutrient management (100% FYM) produced 8.2 and 6.4% more grain and biological yields, respectively over application of chemical fertilizers alone (100% RDF). However, application of chemical fertilizers alone (100% RDF) gave higher net returns (Rs. 64,739 ha^−1^) and benefit cost ratio (2.23) than other treatments mainly due to higher cost of cultivation for the treatments involving FYM. Similarly, reducing the chemical fertilizers to half the recommended dose and supplementing the same quantity through FYM also improved soil properties compared to application of chemical fertilizers alone. Nutrient uptake and N use efficiencies were also markedly improved with MR-6, hence may be more suitable for rainfed conditions of semi-arid region of India. MR-6 also gave higher net returns (Rs. 62,797 ha^−1^) and benefit cost ratio (1.94) than that of MR-1. MR-6 variety can be preferred for cultivation under organic nutrient management (FYM at 8 Mg ha^−1^) for higher productivity. Reduction in chemical fertilizers dose by 50% or completely up to 100% and substituting with organic manures would prove beneficial for sustaining the productivity, soil health and nutrient use efficiency under rainfed conditions.

## Data availability statement

The original contributions presented in the study are included in the article/supplementary material, further inquiries can be directed to the corresponding author.

## Author contributions

MP: conceptualization, resources, and supervision. KG and US: methodology. MT, US, and GSK: formal analysis. GSS: software. MP, KG, and NR: investigation. MT, US, GSK, and GM: data curation. US and GM: writing – original draft preparation. MP, KG, and NR: writing – review and editing. VS: project administration. All authors have read and agreed to the published version of the manuscript.

## References

[1] Gupta SM Arora S Mirza N Pande A Lata C Puranik S. Finger millet: a “certain” crop for an “uncertain” future and a solution to food insecurity and hidden hunger under stressful environments. Front Plant Sci. (2017) 8:643. doi: 10.3389/fpls.2017.00643, PMID: 28487720 PMC5404511

[2] Michaelraj PSJ Shanmugam A. A study on millets-based cultivation and consumption in India. Int J Mark Financ Ser Manag Res. (2013) 2:49–58.

[3] Hema R Vemanna RS Sreeramulu S Reddy CP Senthil-Kumar M Udayakumar M. Stable expression of mtlD gene imparts multiple stress tolerance in finger millet. PLoS One. (2014) 9:e99110. doi: 10.1371/journal.pone.0099110, PMID: 24922513 PMC4055669

[4] Ceasar SA Maharajan T Ajeesh Krishna TP Ramakrishnan M Victor Roch G Satish L. Finger millet [Eleusine coracana (L.) Gaertn.] improvement: current status and future interventions of whole genome sequence. Front. Plant Sci. (2018) 9:1054. doi: 10.3389/fpls.2018.01054, PMID: 30083176 PMC6064933

[5] Rathore T Singh R Kamble DB Upadhyay A Thangalakshmi S. Review on finger millet: processing and value addition. Pharma Innov. (2019) 8:283–91.

[6] Govindappa M Vishwanath AP Harsha KN Thimmegowda P Jnanesh AC. Response of finger millet (Eluesine coracana L.) to organic and inorganic sources of nutrients under rainfed condition. J Crop Weed. (2009) 5:291–3.

[7] GoI. Directorate of Economics and Statistics, Ministry of Agriculture and farmer welfare. New Delhi, India: GoI (2020).

[8] Sakamma S Umesh KB Girish MR Ravi SC Satishkumar M Bellundagi V. Finger millet (Eleusine coracana L. Gaertn.) production system: status, potential, constraints and implications for improving small farmer’s welfare. J Agric Sci. (2018) 10:162–79. doi: 10.5539/jas.v10n1p162

[9] Gull A Jan R Nayik GA Prasad K Kumar P. Significance of finger millet in nutrition, health and value-added products: a review. J Environ Sci Comp Sci Eng Technol. (2014) 3:1601–8.

[10] Maruthi Sankar GR Ravindra Chary G Subba Reddy G GGSN R Ramakrishna YS Girija A. Statistical modeling of rainfall effects for assessing efficiency of fertilizer treatments for sustainable crop productivity under different agro-eco sub-regions. Paper presented at: International symposium on agro-meteorology and food security. Hyderabad: CRIDA (2008).

[11] Mahajan A Bhagat RM Gupta RD. Integrated nutrient management in sustainable rice – wheat cropping system for food security in India. SAARC J Agric. (2008) 6:1–16.

[12] Rao BKR Krishnappa K Srinivasarao C Wani SP Sahrawat KL Pardhasaradhi G. Alleviation of multinutrient deficiency for productivity enhancement of rain-fed soybean and finger millet in the semi-arid region of India. Commun Soil Sci Plant Anal. (2012) 43:1427–35. doi: 10.1080/00103624.2012.670344

[13] Minhas WA Hussain M Mehboob N Nawaz A Ul-Allah S Rizwan MS. Synergetic use of biochar and synthetic nitrogen and phosphorus fertilizers to improves maize productivity and nutrient retention in loamy soil. J Plant Nutr. (2020) 43:1356–68. doi: 10.1080/01904167.2020.1729804

[14] Sravan US Singh SP Neupane MP. Response of basmati rice varieties to integrated nutrient management. J Plant Nutr. (2021) 44:351–65. doi: 10.1080/01904167.2020.1822394

[15] Jackson ML. Soil chemical analysis. New Delhi: Prentice Hall of India Pvt. Ltd (1973).

[16] Ju C Buresh RJ Wang Z Zhang H Liu L Yang J. Root and shoot traits for rice varieties with higher grain yield and higher nitrogen use efficiency at lower nitrogen rates application. Field Crop Res. (2015) 175:47–55. doi: 10.1016/j.fcr.2015.02.007

[17] Walkley AJ Black IA. An examination of the Degtjareff method for determination of soil organic matter and a proposed modification of the chronic acid titration method. Soil Sci. (1934) 37:29–38. doi: 10.1097/00010694-193401000-00003

[18] Subbiah BV Asija GL. A rapid procedure for the estimation of available nitrogen in soils. Curr Sci. (1956) 25:259–60.

[19] Olsen SR Cole CV Watanabe FS Dean LA. Estimation of available phosphorus in soil by extraction with sodium bicarbonate. (1954) Washington, DC: USDA, Circular number 939:1–19.

[20] Chesnin L Yien CH. Turbidimetric determination of available sulphate. Soil Sci Soc Amer Proc. (1951) 15:149–51. doi: 10.2136/sssaj1951.036159950015000C0032x

[21] Lindsay WL Norvell WA. Development of a DTPA soil test for zinc, iron, manganese and copper. Soil Sci Soc Am J. (1978) 42:421–8. doi: 10.2136/sssaj1978.03615995004200030009x

[22] Archer DW Jaradat AA Johnson JMF Weyers SL Gesch RW Forcella F. Crop productivity and economics during the transition to alternative cropping systems. Agron J. (2007) 99:1538–47. doi: 10.2134/agronj2006.0364

[23] De Mendiburu F. Agricolae: Statistical procedures for agricultural research. R package version 1.3–1. (2019) Available at: https://CRAN.R-project.org/package=agricolae

[24] Chohan MSM Naeem M Khan AH Kainth RA. Performance of pearl millet (*Pennisetum americanum* L.) varieties for forage yield. J Agric Res. (2006) 44:23–7.

[25] Faridullah AA Irshad M Khan J Khan AR Sher H Khan K. Comparative studies of different pearl millet varieties as affected by different yield components. Elec J Env Agricult Food Chem. (2010) 9:1524–33.

[26] Triveni U Sandhya Rani Y Patro TSSK Anuradha N Divya M. Response of improved long duration finger millet (*Eleusine coracana* L.) genotypes to different levels of NPK fertilizers under rainfed conditions. Progressive Res Int J. (2017) 12:22–6.

[27] Triveni U Sandhya Rani Y Patro TSSK Anuradha N Divya M. Fertilizer responsiveness of short duration improved finger millet genotypes to different levels of NPK fertilizers. Indian J Agric Res. (2018) 52:97–100. doi: 10.18805/IJARe.A-4801

[28] Saha S Ved PC Kundu S Kumar N Mina B. Soil enzymatic activity as affected by long term application of farm yard manure and mineral fertilizer under a rainfed soybean-wheat system in N-W Himalaya. Eur J Soil Biol. (2008) 44:309–15. doi: 10.1016/j.ejsobi.2008.02.004

[29] Sunitha N Ravi V Reddy R. Nitrogen economy in finger millet through conjunctive use of organic manures and bio-fertilizers. Indian J Agron. (2006) 21:96–8.

[30] Maitra S Devender Reddy M Nanda SP. Nutrient management in finger millet (Eleusine coracana L. Gaertn) in India. Int J Agric Environ Biotechnol. (2020) 13:3–21. doi: 10.30954/0974-1712.1.2020.2

[31] Prabhakar Ganiger PC Boraiah B Bhat Sujata Nandini C Kiran Tippeswamy V Manjunath HA. Improved production technologies for finger millet. ICAR-AICRP on Small Millets: GKVK, Bengaluru, Technical Bulletin 1/2017-18 (2017). 32.

[32] Arulmozhiselvan KM Elayarajan SS. Effect of long-term fertilization and manuring on soil fertility, yield and uptake by finger millet on inceptisol. Madras Agric J. (2013) 100:490–4.

[33] Aravind SA Senthil Kumar N Hemalatha M Paramasivan M. Impact on soil health under organic nutrient management in transplanted finger millet (Eleusine coracana L.). Int J Plant Soil Sci. (2022) 34:1399–406. doi: 10.9734/ijpss/2022/v34i2231512

[34] Prashanth DV Krishnamurthy R Naveen DV. Long-term effect of integrated nutrient management on soil nutrient status, content and uptake by finger millet crop in a Typic Kandiustalf of eastern dry zone of Karnataka. Commun Soil Sci Plant Anal. (2019) 51:161–74. doi: 10.1080/00103624.2019.1695829

[35] Zerihun A Sharma JJ Nigussie D Fred K. The effect of integrated organic and inorganic fertilizer rates on performances of soybean and maize component crops of a soybean/maize mixture at Bako, Western Ethiopia. Afr J Agric Res. (2013) 8:3921–9. doi: 10.5897/AJAR12.1044

[36] Chakraborty T Roy DK Sounda G. Effect of fertilizer, rock phosphate and Azospirillum on growth and yield of finger millet (Eleusine coracana L. Gaertn). Indian J Agric Res. (2002) 36:192–5.

[37] Wang XJ Jia ZK Liang LY Kang SZ. Effect of manure management on the temporal variations of dryland soil moisture and water use efficiency of maize. J Agric Sci Technol. (2013) 15:1293–304.

[38] Gan YT Campbell CA Janzen HH Lemke R Liu LP Basnyat P. Root mass for oilseed and pulse crops: growth and distribution in the soil profile. Can J Plant Sci. (2009) 89:883–93. doi: 10.4141/CJPS08154

[39] Wafula WN Korir NK Ojulong HF Siambi M Gweyi-Onyango JP. Phosphorus influence on plant tissue nitrogen contents and yield attributes of finger millet varieties in semi-arid region of Kenya. Int J Plant Soil Sci. (2016) 13:1–9. doi: 10.9734/IJPSS/2016/29901

[40] Chowdary KA Patra BC. Effect of micronutrient application with different sources of npk on growth and yield of finger millet crop in red laterite zone. J Agric Sci Technol. (2019) 9:403–16. doi: 10.17265/2161-6264/2019.06.004

[41] Shankar AG Udayakumar M Prasad TG. Genotypic variability for net photosynthesis in finger millet (*Eleusine coracana* G.) genotypes: an approach to identity high CER types. J Agron Crop Sci. (1990) 165:240–52. doi: 10.1111/j.1439-037X.1990.tb00858.x

[42] Garnett T Conn V Kaiser BN. Root based approaches to improving nitrogen use efficiency in plants. Plant Cell Environ. (2009) 32:1272–83. doi: 10.1111/j.1365-3040.2009.02011.x 19558408

[43] Gupta N Gupta AK Gaur VS Kumar A. Relationship of nitrogen use efficiency with the activities of enzymes involved in nitrogen uptake and assimilation of finger millet genotypes grown under different nitrogen inputs. Sci World J. (2012) 2012:625731. doi: 10.1100/2012/625731, PMID: 22919342 PMC3415157

[44] Singh U Ladha JK Castillo EG Punzalan G TirolPadre A Duqueza M. Genotypic variation in nitrogen use efficiency in medium- and long-duration rice. Field Crop Res. (1998) 58:35–53. doi: 10.1016/S0378-4290(98)00084-7

[45] Bowen GD Rovira AD. The rhizosphere and its management to improve plant growth. Adv Agron. (1999) 66:1–102. doi: 10.1016/S0065-2113(08)60425-3

[46] Jagathjothi N Ramamoorthy K Sathya PR. Influence of enriched FYM with inorganic fertilizers on nutrient uptake, soil available nutrients and productivity of rainfed finger millet. Madras Agric J. (2010) 97:385–7.

[47] Sharma S Chander G Verma TS Sudhir V. Soil potassium fractions in rice-wheat cropping system after twelve years of lantana residue incorporation in a northwest Himalayan acid Alfisol. J Plant Nutr. (2013) 36:1809–20. doi: 10.1080/01904167.2013.815202

[48] Vineela C Wani SP Srinivasarao C Padmaja B Vittal KPR. Microbial properties of soil as affected by cropping and nutrient management practices in several long-term manorial experiments in the semi-arid tropics of India. Appl Soil Ecol. (2008) 40:165–73. doi: 10.1016/j.apsoil.2008.04.001

[49] Srinivasarao C Venkateswarlu B Lal R Singh AK Kundu S. Sustainable management of soils of dryland ecosystems of India for enhancing agronomic productivity and sequestering carbon. Adv Agron. (2013) 121:253–329. doi: 10.1016/B978-0-12-407685-3.00005-0

[50] Srinivasarao C Venkateswarlu B Singh AK Vittal KPR Kundu S Gajanan GN. Yield sustainability and carbon sequestration potential of groundnut finger millet rotation in alfisols under semi-arid tropical India. Int J Agric Sustain. (2012) 10:230–44. doi: 10.1080/14735903.2012.662392

[51] Prakash YS Bhadoria PBS Varakshit A. Comparative efficiency of organic manures on the changes in soil properties and nutrient availability in an Alfisol. J Indian Soc Soil Sci. (2003) 50:219–21.

[52] Tolanur SI Badanur VP. Changes in organic carbon, available N, P and K under integrated use of organic manure, green manure and fertilizers on sustaining productivity of pearl millet pigeonpea system and fertility of an Inceptisol. J Indian Soc Soil Sci. (2003) 51:254–7.

[53] Roy SK Sharma RC Trehan SP. Integrated nutrient management by using farmyard manure and fertilizers in potato-sunflower-paddy rice rotation in the Punjab. J Agric Sci. (2001) 137:271–8. doi: 10.1017/S0021859601001472

[54] Jaskulska I Jaskulski D Kobierski M. Effect of liming on the change of some agrochemical soil properties in a long-term fertilization experiment. Plant Soil Environ. (2014) 60:146–50. doi: 10.17221/PSE

[55] Sharma RP Datt N Chander G. Effect of vermicompost, farmyard manure and chemical fertilizers on yield, nutrient uptake and soil fertility in okra (*Abelmoschus esculentus*) –onion (*Allium cepa*) sequence in wet temperate zone of Himachal Pradesh. J Indian Soc Soil Sci. (2009) 57:357–61.

[56] Swarup A. Effect of micronutrient and farmyard manure on the yield and micronutrient of rice and wheat grown on a sodic soil. J Ind Soc Soil Sci. (1984) 32:397–9.

[57] Chaudhary M Narwal RP. Effect of long-term application of farmyard manure on soil micronutrients status. Arch Agron Soil Sci. (2005) 51:351–9. doi: 10.1080/03650340500133134

[58] Antil RS Singh M. Effects of organic manures and fertilizers on organic matter and nutrients status of the soil. Arch Agron Soil Sci. (2007) 53:519–28. doi: 10.1080/03650340701571033

[59] Satish A Ramachandrappa BK Shankar MA Srikanth Babu PN Srinivasarao C Sharma KL. Long-term effects of organic manure and manufactured fertilizer additions on soil quality and sustainable productivity of finger millet under a finger millet–groundnut cropping system in southern India. Soil Use Manag. (2016) 32:311–21. doi: 10.1111/sum.12277

